# The Antimicrobial Peptide Mastoparan X Protects Against Enterohemorrhagic *Escherichia coli* O157:H7 Infection, Inhibits Inflammation, and Enhances the Intestinal Epithelial Barrier

**DOI:** 10.3389/fmicb.2021.644887

**Published:** 2021-06-10

**Authors:** Xueqin Zhao, Lei Wang, Chunling Zhu, Xiaojing Xia, Shouping Zhang, Yimin Wang, Huihui Zhang, Yanzhao Xu, Shijun Chen, Jinqing Jiang, Shanqin Liu, Yundi Wu, Xilong Wu, Gaiping Zhang, Yueyu Bai, Hanna Fotina, Jianhe Hu

**Affiliations:** ^1^College of Animal Science and Veterinary Medicine, Henan Institute of Science and Technology, Xinxiang, China; ^2^Faculty of Veterinary Medicine, Sumy National Agrarian University, Sumy, Ukraine; ^3^State Key Laboratory of Marine Resource Utilization in South China Sea, School of Biomedical Engineering, Hainan University, Haikou, China; ^4^School of Chemistry and Chemical Engineering, Henan Institute of Science and Technology, Xinxiang, China; ^5^College of Animal Science and Veterinary Medicine, Henan Agricultural University, Zhengzhou, China

**Keywords:** antimicrobial peptide mastoparan X, inflammation, intestinal barrier, Enterohemorrhagic *Escherichia coli* O157:H7, mice

## Abstract

*Escherichia coli* can cause intestinal diseases in humans and livestock, destroy the intestinal barrier, exacerbate systemic inflammation, and seriously threaten human health and animal husbandry development. The aim of this study was to investigate whether the antimicrobial peptide mastoparan X (MPX) was effective against *E. coli* infection. BALB/c mice infected with *E. coli* by intraperitoneal injection, which represents a sepsis model. In this study, MPX exhibited no toxicity in IPEC-J2 cells and notably suppressed the levels of interleukin-6 (IL-6), tumor necrosis factor-alpha (TNF-α), myeloperoxidase (MPO), and lactate dehydrogenase (LDH) released by *E. coli*. In addition, MPX improved the expression of ZO-1, occludin, and claudin and enhanced the wound healing of IPEC-J2 cells. The therapeutic effect of MPX was evaluated in a murine model, revealing that it protected mice from lethal *E. coli* infection. Furthermore, MPX increased the length of villi and reduced the infiltration of inflammatory cells into the jejunum. SEM and TEM analyses showed that MPX effectively ameliorated the jejunum damage caused by *E. coli* and increased the number and length of microvilli. In addition, MPX decreased the expression of IL-2, IL-6, TNF-α, p-p38, and p-p65 in the jejunum and colon. Moreover, MPX increased the expression of ZO-1, occludin, and MUC2 in the jejunum and colon, improved the function of the intestinal barrier, and promoted the absorption of nutrients. This study suggests that MPX is an effective therapeutic agent for *E. coli* infection and other intestinal diseases, laying the foundation for the development of new drugs for bacterial infections.

## Introduction

*Escherichia coli* is a facultative anaerobe that causes diarrhea, enteritis, host intestinal barrier damage, and intestinal microecological disorders, which have high mortality rates and result in substantial losses worldwide (Glombowsky et al., 2020). *Escherichia coli* can also induce urinary tract infections and meningitis in neonates (Chen et al., 2020). Although antibiotics are currently effective in treating *E. coli* infections, diseases caused by *E. coli* have high degrees of sequelae (Ding et al., 2020). Antibiotics are the primary treatment for many pathogenic *E. coli* strains infection in animal production, leading to increased resistance to antibiotics, such as ampicillin, florfenicol, and gentamicin (Smith et al., 2016). The number of published surveys on resistance to *E. coli* in LMICs increased from 3 in 2000 to 121 in 2018 and peaked at 156 per year in 2017. The highest resistance rates were observed in the most commonly used classes of antimicrobials in animal production, tetracyclines, sulfonamides, and penicillins (Van Boeckel et al., 2019). Thus, there is a dire need to explore new antibiotic alternatives to resist *E. coli* infections.

As we know, the high similarity between pigs and humans makes pigs a good gastrointestinal (GI) model for humans, the advantages of using IPEC-J2 as *in vitro* model of the GI tract are the high resemblance between humans and pigs (Geens and Niewold, 2011). IPEC-J2 cells maintain their differentiated characteristics and exhibit strong similarities to primary intestinal epithelial cells and can be an appropriate model through the advantage of direct comparison with the experimental animal, which in turn might serve as a good model for humans, demonstrating that IPEC-J2 cells represent a better model of normal intestinal epithelial cells than transformed cell lines (Schierack et al., 2006). In addition, a number of data demonstrate the functional resemblance of IPEC-J2 to the well-defined human colon carcinoma cell line Caco-2 (Pinto et al., 1983). Therefore, IPEC-J2 was selected for this study.

Due to the alarming increase in pathogen resistance to conventional antibiotics and threats to public health worldwide, the exploration of new drugs to treat infections caused by antimicrobial-resistant pathogens is urgently needed (Alfei and Schito, 2020). Antimicrobial peptides are a class of small molecular peptides produced by the bodily innate immune system that can resist pathogenic infection and exhibit antibacterial, antiviral, anti-inflammatory, and immunoregulatory functions (Faya et al., 2020; Lee et al., 2020). Antimicrobial peptides are currently considered as the best alternatives to antibiotics. The antimicrobial peptide mastoparan X (MPX) extracted from venom contains both acidic and basic residues (including three basic residues), possesses a net charge of 4, and has good antibacterial activity against Gram-positive and Gram-negative microorganisms (Leite et al., 2011). The action mode of MPX is associated with the peptide hydrophobicity and electrostatic interactions between the positive charges of the peptides and the negative charges of the phosphate group of cell membrane phospholipids (Da Silva et al., 2018). Henriksen et al. (2014) found that modulating peptide hydrophobicity by introducing an unnatural amino acid with an octyl side chain *via* amino acid substitution at positions 1, 8, and 14 increased the bactericidal potency of the antimicrobial peptide MPX (Henriksen et al., 2014). We previously showed that MPX had good antimicrobial activity against *Actinobacillus pleuropneumoniae* infections, reduced the colonization of *A. pleuropneumoniae* in the lungs, alleviated the symptoms of pneumonia, and improved the survival rate of mice (Wang et al., 2020). However, the effects of MPX on *E. coli* infections and on the regulation of *E. coli*-induced inflammation and intestinal disruption remain unknown.

In this study, the effects of MPX on *E. coli*-induced intestinal inflammation and barrier dysfunction were investigated *in vitro* and *in vivo*. In IPEC-J2 cells, MPX exhibited no cytotoxicity and reduced the mRNA expression of interleukin (IL)-6 and tumor necrosis factor-alpha (TNF-α) by inhibiting the phosphorylation of p38 and p65. In addition, MPX increased the *E. coli*-induced expression of the tight junction (TJ) proteins ZO-1 and occludin and promoted the healing of intestinal epithelial cell damage. These results were further confirmed *in vivo*, *E. coli*-infected mice by intraperitoneal injection, while not representative for intestinal infection with the used *E. coli* strain, as MPX protected against lethal *E. coli* infection, improved the survival rate of mice, alleviated intestinal inflammation in the jejunum and colon by reducing the expression of inflammatory factors, and increased the expression of TJ proteins and the number of microvilli, thereby improving intestinal barrier function. These findings suggest a promising protective role of MPX in preventing *E. coli* infections, laying the foundation for the development of alternatives to conventional antibiotics.

## Materials and Methods

### Ethics Statement

All animal experiments were approved by the Animal Ethics Committee of Zhengzhou University and were performed in accordance with the guidelines of the Animal Welfare and Research Ethics Committee.

### Peptide Synthesis

MPX (H-INWKGIAAMAKKLL-NH2) was synthesized and purified by Ji er sheng hua (Shanghai, China) at purity greater than 98%, as determined by high-performance liquid chromatography (HPLC) and mass spectrometry. MPX was dissolved in ddH_2_O and stored at −20°C.

### Preparation of the *Escherichia coli* Strain

*Escherichia coli* (Enterohemorrhagic *Escherichia coli* O157:H7 ATCC43889) were isolated from feces of patient with hemolytic uremic syndrome and obtained from the China Institute of Veterinary Drug Control (Beijing, China). The *E. coli* strain was seeded on LB (Solarbio, China) agar to obtain isolated pure colonies, and a single colony was inoculated into LB broth and incubated overnight at 37°C while shaking at 180 rpm (75003442, Thermo, United States) for 10 h. In order to get the bacteria to the logarithmic growth phase, 100 μl was transferred into 10 ml of fresh LB broth and incubated at 37°C while shaking at 180 rpm for 4 h. Then, 1 ml of *E. coli* bacteria liquid in a 1.5-ml centrifuge tube, centrifuged at 8,000 rpm (6,010 g) for 5 min, and resuspended the bacterial pellet with phosphate buffer (pH = 7.4). The *E. coli* bacteria liquid was then serially diluted 10 times, seeded onto LB plates and placed the LB plates in a 37°C incubator for 12 h, and counted the number of *E. coli* per milliliter after growing visible single bacteria. The result was 4.5 × 10^8^ CFU/ml. The bacteria were diluted by decimal serial dilutions one time, and 4.5 × 10^7^ CFU/ml was obtained.

### Cell Culture

The porcine intestinal epithelial cell line IPEC-J2 is intestinal columnar epithelial cells, which were isolated from neonatal piglet midjejunum, and donated by professor Liancheng Lei of Jilin University. IPEC-J2 cells were cultured in DMEM supplemented with 10% fetal bovine serum and 1% antibiotics (penicillin and streptomycin) and incubated at 37°C and 5% CO_2_. When they reached 100% confluence, the cells were cultured in DMEM without 1% antibiotics and then treated with MPX (10 μg/ml) for 2 h. Then, the cells were further cultured with *E. coli* (MOI = 10) for 12 h.

### Cytotoxicity Studies

Cell viability was determined by the CCK-8 kit (Meilunbio, China) in accordance with the manufacturer’s instructions. First, IPEC-J2 cells were cultured in 96-well plates at 1 × 10^4^ cells/well and treated with the indicated concentration (2–512 μg/ml) of MPX for 24 h. Then, the IPEC-J2 cells were incubated with 10 μl of CCK-8 per well in a cell culture incubator for 2 h, and the absorbance of each well was detected at 450 nm with a microplate reader (Dynatech Laboratories, United States).

The lactose dehydrogenase (LDH) release assay (Nanjing Jiancheng, China) was performed to determine whether MPX damaged the IPEC-J2 cell membrane in accordance with the manufacturer’s instructions. Briefly, IPEC-J2 cells were cultured in 96-well plates at 1 × 10^4^ cells/well and treated with the indicated concentration (2–512 μg/ml) of MPX at 37°C and 5% CO_2_ for 24 h. In addition, the IPEC-J2 cells were pretreated with MPX (10 μg/ml) for 2 h and infected with *E. coli* (MOI = 10) for different amounts of time (3, 6, 12, and 24 h). The optical densities were measured at 450 nm using a microplate reader (VARIOSKAN FLASH, United States).

### Wound Healing Assay

IPEC-J2 cells were cultured in 6-well plates, and the cell monolayer was scraped with a 200-μL pipette tip. The recovered IPEC-J2 cells were incubated with medium only or with 10 μg/ml MPX for different amounts of time, and images were acquired at 0, 48, and 96 h by Nikon (Ti-E 531235, Japan). The wound width was measured at 48 h by ImageJ2x (Rawak Software Inc., Germany):Rawak Software Inc., Germany, and each sample was analyzed in triplicate.

### Trans-Epithelium Electrical Resistance Measurements

IPEC-J2 cells were seeded onto polycarbonate membrane filters (0.4-μm pore size, 6.5 mm Diameter Inserts) inside Transwell® cell culture chambers (Corning Incorporated costar, United States) at a density of 1 × 10^5^ cells/cm^2^. The culture medium was changed every day and trans-epithelium electrical resistance (TEER) values were measured every other day using the world precision instruments (EVOM^2^, United States). When a monolayer of cells was considered to be completely differentiated, cells were treated with PBS, *E. coli*, MPX, *E. coli* + MPX (cells were treated with *E. coli* (MOI = 1), followed by MPX) and the TEER was measured at 0, 4, 8, 12, 16, 20, and 24 h, and there were three replicates for each experiment.

### Real-Time PCR

The primer sequences for real-time PCR are shown in Table 1. Total RNA was extracted using RNA extraction kit reagent (Solarbio, China). RNA concentration and OD260/OD280 were measured by NanoDrop 2000 spectrophotometer (Thermo Fisher Scientific, United States). The integrity of RNA was verified by visualization in an agarose gel. The RIN of samples was 9. In addition, 2 μg of total RNA was converted to cDNA. cDNA was obtained using a reverse transcription kit (Thermo Scientific, United States). Each reaction (10 μl) volume included 5 μl of SYBR Green Master Mix (QuantiNova, China), 0.5 μl of the forward primer (10 μM), 0.5 μl of the reverse primer (10 μM), 0.5 μl of cDNA, and 3.5 μl of ddH_2_O. The thermocycler reaction included 2 min at 95°C and 40 cycles of 20 s at 95°C and 30 s at 60°C, and melt curves were added; GAPDH served as the housekeeping gene. The 2^−ΔΔCt^ method was used to calculate the relative mRNA expression levels (Omonijo et al., 2018).

**Table 1:** The primer sequences for real-time PCR.

Genes	Sequence
MUC2 (mouse)	F:5'-CTGCTCCGGGTCCTGTGGGA-3'
	R:5'-CCCGCTGGCTGGTGCGATAC-3'
TNF-α (mouse)	F:5'-CTCATGCACCACCATCAAGG-3'
	R:5'-ACCTGACCACTCTCCCTTTG-3'
IL-6 (mouse)	F:5'-CTCTGGCGGAGCTATTGAGA-3'
	R:5'-AAGTCTCCTGCGTGGAGAAA-3'
IL-2 (mouse)	F:5'-CCTGAGCAGGATGGAGAATTACA-3'
	R:5'-TCCAGAACATGCCGCAGAG-3'
Occludin (mouse)	F:5'-ACGGACCCTGACCACTATGA-3'
	R:5'-TCAGCAGCAGCCATGTACTC-3'
Claudin-1 (mouse)	F:5'-AGCTGCCTGTTCCATGTACT-3'
	R:5'-CTCCCATTTGTCTGCTGCTC-3'
ZO-1 (mouse)	F:5'-ACCCGAAACTGATGCTGTGGATAG-3'
	R:5'-AAATGGCCGGGCAGAACTTGTGTA-3'
GAPDH (mouse)	F:5'-TGGAGAAACCTGCCAAGTATGA-3'
	R:5'-TGGAAGAATGGGAGTTGCTGT-3'
IL-6 (pig)	F:5'-TGGCTACTGCCTTCCCTACC-3'
	R:5'-CAGAGATTTTGCCGAGGATG-3'
TNF-α (pig)	F:5'-ATGGATGGGTGGATGAGAAA-3'
	R:5'-TGGAAACTGTTGGGGAGAAG-3'
Claudin-1 (pig)	F:5'-CCATCGTCAGCACCGCACTG-3'
	R:5'-CGACACGCAGGACATCCACAG-3'
Occludin (pig)	F:5'-GACAGACTACACAACTGGCGG-3'
	R:5'-TGTACTCCTGCAGGCCACTG-3'
ZO-1 (pig)	F:5'-ATGAGCAGGTCCCGTCCCAAG-3'
	R:5'-GGCGGAGGCAGCGGTTTG-3'
GAPDH (pig)	F:5'-ACTCACTCTTCCACTTTTGATGCT-3'
	R:5'-TGTTGCTGTAGCCAAATTCA-3'

### Western Blotting

Total protein was extracted with RIPA lysate buffer (KeyGEN, China), and the protein concentration was determined using a BCA protein content kit (Biyuntian, China). Then, 20 μg of protein extracted from the lysate supernatant was separated by 10% SDS-PAGE and transferred onto a PVDF membrane. After blocking with 5% bovine serum albumin (BSA) for 2 h, a rabbit anti-occludin monoclonal antibody (1:1,000 dilution, ab31721, Abcam) incubated with PVDF membrane overnight at 4°C and washed five times with TBST for 7 min each time. The PVDF membrane was then incubated with a goat anti-rabbit horseradish peroxidase (HRP)-conjugated secondary antibody rabbit anti-β-actin monoclonal antibody (1:1,000 dilution, #4970, Cell Signaling Technology) for 1 h at room temperature and washed five times with TBST for 7 min each time (Xin et al., 2019). The bands were detected using ECL (Solarbio, China), and the band intensities were quantified by ImageJ software.

### Transmission Electron Microscopy

The TJs and microvillus morphologies of mouse intestinal epithelial cells were observed by transmission electron microscopy (TEM; Narayana et al., 2015). Mouse jejunum specimens were obtained with a scalpel and fixed in 2.5% glutaraldehyde for 12 h at 4°C. Then, the jejunum samples were treated with osmic acid and embedded in Epon, and ultrathin sections were acquired using a diamond knife and then stained with uranyl acetate and lead citrate before being observed by TEM (7610plus/FEI Apreo, Japan).

### Scanning Electron Microscopy

The morphologies of the mouse jejunum villi and microvilli were observed by scanning electron microscopy (SEM; Yi et al., 2016). Jejunum tissues from the mice were fixed with 2.5% glutaraldehyde overnight at 4°C and then incubated with 1% OsO4 for 1 h. The jejunum specimens were then dehydrated with an ethanol gradient (30, 50, 70, 80, 90, 95, and 100%) for 15 min at each step and treated with a mixture of alcohol and isoamyl acetate (v:v = 1:1) for 30 min. Then, isoamyl acetate was added for 1 h, and the dehydrated specimens were coated with gold–palladium and visualized with a Philips Model SU8010 FASEM (HITACHI, Japan).

### ELISA

The serum levels of IL-6, TNF-α, and IL-2 were determined using ELISA kits (Biolegend, United States). In addition, the serum level of myeloperoxidase (MPO) was detected using an ELISA kit (Multi Science, China). All samples were measured according to the manufacturers’ instructions.

### Histopathology and Immunohistochemistry

The jejunum and colon tissues of the mice were fixed in 4% paraformaldehyde for 24 h and embedded in paraffin. Hematoxylin and eosin (H&E) staining was used to stain the jejunum, and images were obtained using a DM3000 microscope (PHASE CONTRAST, Japan). Image-Pro software (ipwin32 software; Media Cybernetics, United States) was used to measure the villous height and crypt depth, and the ratio of villous height to crypt depth was then calculated (Wang et al., 2017).

For immunohistochemistry (IHC), 5-μm-thick sections of the jejunum and colon tissues were embedded in paraffin, incubated in sodium citrate buffer (pH = 6.0) to repair the antigens and placed in a 3% hydrogen peroxide solution to block endogenous peroxidase after dewaxing and rehydration. To block nonspecific binding sites, the sections were incubated with 3% BSA for 30 min and then incubated with rabbit anti-p-p38 (1:200 dilution, #4511, Cell Signaling Technology), rabbit anti-p-pERK (1:200 dilution, #4370, Cell Signaling Technology), and rabbit and anti-p-pJNK antibodies (1:200 dilution, #4668, Cell Signaling Technology) overnight at 4°C. Then, the sections were incubated with HRP-conjugated secondary antibodies (1:200 dilution, #7074, Cell Signaling Technology) for 50 min and counterstained with hematoxylin after development with DAB buffer (Zhang et al., 2019).

### Immunofluorescence

IPEC-J2 cells were cultured on plates especially designed for confocal laser microscopy, pretreated with MPX (10 μg/ml) for 2 h and then incubated with *E. coli* (MOI = 10) at 37°C for 12 h. Then, the IPEC-J2 cells were washed three times with PBS (pH = 7.4) and fixed with 4% paraformaldehyde for 15 min. The IPEC-J2 cells were washed three times with PBS (pH = 7.4), blocked with 1% BSA at room temperature for 30 min, incubated with primary rabbit antibodies overnight at 4°C (rabbit anti-occludin (1:200 dilutions, abs136990, absin)), anti-p-p38 (1:1,600 dilutions, #4511s, Cell Signaling Technology), anti-p-p65 (1:1,600 dilutions, #3033s, Cell Signaling Technology), and anti-TLR4 (1:500 dilutions, bs-20594R, Bioss) incubated with the secondary antibody (Alexa Fluor® 488-anti-rabbit; 1:200 dilutions, ZF-0511, ZSGB-BIO) for 1 h and stained with DAPI for 15 min. TJ and inflammatory proteins were observed under a confocal laser microscope (EVOS M7000, United States; Chromek et al., 2012). In addition, MUC2 (1:200 dilutions, GB11334, Servicebio), p-p65 (1:100 dilutions, GB13025-1, Servicebio), ZO-1 (1:300 dilutions, GB11195, Servicebio), claudin-1(1:200 dilutions, GB11032, Servicebio), occludin (1:200 dilutions, GB11149, Servicebio), and goat anti-rabbit label CY3 (1:300 dilutions, GB21303, Servicebio). These antibodies were used in the immunofluorescence staining of mouse jejunum and colon.

### Animals and Sample Collection

A total of 20 BALB/c mice (6–8 weeks old, 18–20 g, female) were purchased from Zhengzhou University. After the cages were cleaned and disinfected, all mice were housed in individual cages at a constant humidity (40–70%) and temperature (21 ± 1°C) under a 12-h light/dark cycle for 3 days to acclimate to the environment. The mice had *ad libitum* access to food and water before the start of the experiment. The animals were randomly divided into four experimental groups (control, *E. coli*, *E. coli* + MPX, and *E. coli* + enrofloxacin (Enro); five mice per group) and challenged with an intraperitoneal injection of *E. coli* (4.5 × 10^7^ CFU/ml; Ribes et al., 2020). The mice were treated with an intraperitoneal injection of MPX (20 mg/kg) or Enro (20 mg/kg) for 3 days after *E. coli* infection for 2 h. The clinical symptoms of the mice, including their fur state, body weight changes, mental state, and appetite, were recorded every day. The specific scoring criteria were as follows: no clinical signs, 0; slight clinical signs, 1; moderate clinical signs, 2; and severe clinical signs, 3. The mouse sera were used for inflammatory factor analysis, and their livers, spleens, lungs, and intestines were collected and fixed with 4% paraformaldehyde for H&E staining, immunohistochemistry, and immunofluorescence analysis. The jejunum samples were fixed with glutaraldehyde (2.5%) to observe changes in the intestinal villi and microvilli by SEM and TEM.

### Fecal and Tissue Microbiota Counts

The mice were euthanized, and their feces and tissues were collected aseptically. The fecal and tissue microbiota counts were determined and calculated by the dilution counting method in a sterile room. The fresh fecal samples were resuspended in sterile PBS and vortexed (0.5 g of fresh feces in 4.5 ml of sterile saline), and appropriate 100 μl dilutions of fecal tissues were spread over MacConkey agar plates to count the colonies. In addition, different volumes of PBS were added according to the tissue weights, and appropriate 100 μl dilutions of tissues were spread over MacConkey agar plates to count the colonies. The plates were incubated in a 37°C incubator for 12 h under anaerobic conditions, and the results are shown as CFU/g feces and tissues. The assays were repeated three times.

### Statistical Analysis

Statistical analyses were performed using GraphPad Prism software (version 8.0, La Jolla, CA, United States). All the results are expressed as the mean ± SEM. Group comparisons were performed by one-way analysis of variance (ANOVA) followed by Tukey’s test. Statistical significance was expressed as *p* < 0.05 as follows: *p < 0.05; **p < 0.01; ***p < 0.001; ^#^*p* < 0.05, ^##^*p* < 0.01, and ^###^*p* < 0.001.

## Results

### MPX Reduces LDH Release and Inhibits Inflammatory Cytokine Expression

**Figure 1:**
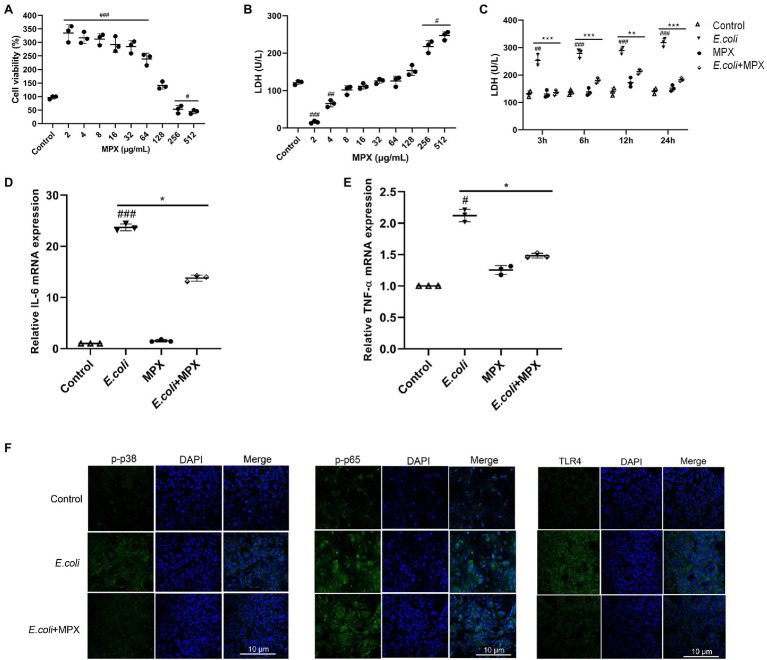
MPX does not induce cytotoxicity and alleviates inflammation in IPEC-J2 cells. (A) Cell viability was measured using the CCK-8 assay, IPEC-J2 cells were cultured with different concentrations (2-512 µg/mL) of MPX for 24 h. (B) The release of LDH from IPEC-J2 cells after treatment with different concentrations (2-512 µg/mL) of MPX for 24 h. (C) MPX decreased the E. coli-induced release of LDH from IPEC-J2 cells (MOI = 10) at different times. (D,E) The mRNA expression of IL-6 and TNF-α after MPX treatment. (F) The expression of p-p38, p-p65 and TLR4 in IPEC-J2 cells assessed by confocal laser microscopy. Error bars represent mean ± SEM, n = 3. Statistical significance was defined as follows: #P < 0.05; ##P < 0.01; ###P < 0.001 E. coli vs control; *P < 0.05; **P < 0.01; ***P < 0.001 MPX treatment vs E. coli.

The cytotoxicity of MPX was assessed since this study aimed to develop this peptide as a safe alternative to antibiotics. A CCK-8 kit was used to determine the viability of IPEC-J2 cells after treatment with MPX at different concentrations (2–512 μg/ml) for 24 h. Compared with the control treatment, the results showed that cell viability analyzed by the CCK-8 assay was not affected, but it might increase cellular metabolism when cells were supplemented with MPX (Figure 1A). The effect on cultured cells was not significant, even at a high concentration of 128 μg/ml. The release of LDH was examined to further determine the toxicity of MPX. Compared with the control group, treatment with different concentrations (2–512 μg/ml) of MPX for 24 h did not significantly increase the release of LDH, even at a concentration of 128 μg/ml (Figure 1B). Moreover, the LDH release from IPEC-J2 cells was notably reduced after pretreatment with MPX for 2 h prior to infection with *E. coli* (Figure 1C, *p* < 0.05). These results indicated that MPX maintained the cellular membrane integrity of IPEC-J2 cells.

The increased expression of inflammatory factors, such as IL-2, IL-6, and TNF-α, is closely related to the inflammatory response (Millot et al., 2020). To evaluate the anti-inflammatory effects of MPX after *E. coli* infection, the expression of IL-6 and TNF-α was determined by real-time PCR. Compared with *E. coli* alone, treatment with MPX significantly inhibited the *E. coli*-induced mRNA expression of IL-6 and TNF-α (Figures 1D,E, *p* < 0.05). In addition, the confocal laser microscopy results showed that *E. coli* infection significantly increased the expression of p-p38, p-p65, and TLR4, while pretreatment with MPX significantly decreased the expression of p-p38, p-p65, and TLR4 (Figure 1F), indicating that MPX could inhibit the release of inflammatory cytokines by reducing the phosphorylation of p38 and the activation of p65 and TLR4.

### MPX Inhibits *Escherichia coli*-Induced Tight Junction Damage in IPEC-J2 Cells

**Figure 2:**
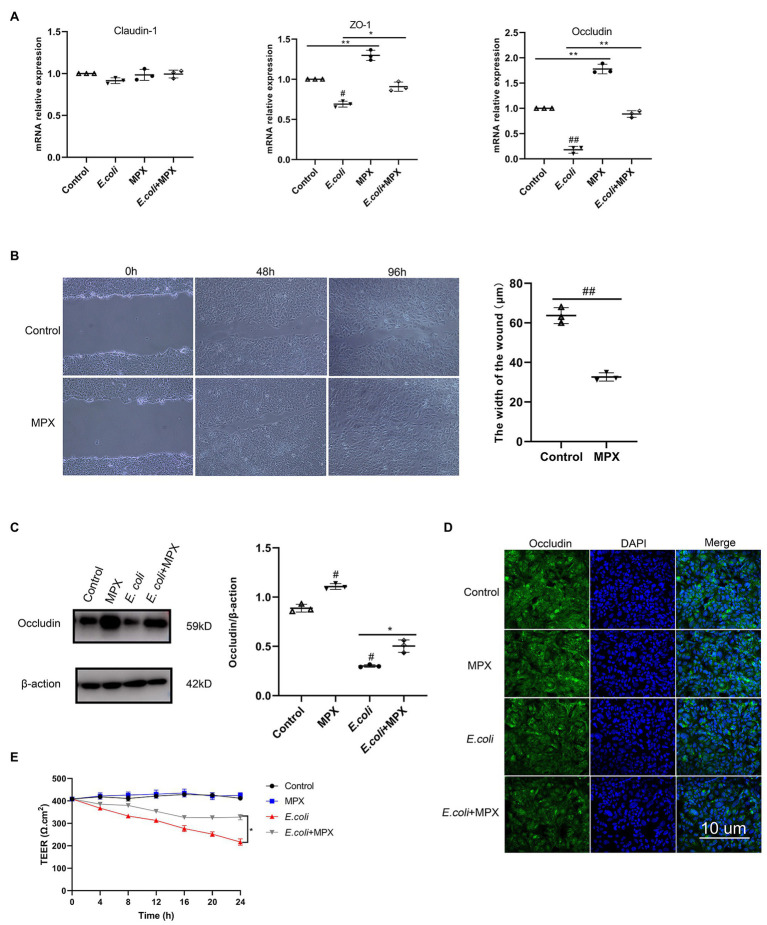
MPX enhances IPEC-J2 cell barrier function. (A) The E. coli-induced mRNA expression of ZO-1, occludin and claudin-1 in IPEC-J2 cells after treatment with MPX. (B) IPEC-J2 cells were incubated with medium alone or MPX (10 μg/mL) in a wound healing assay. Images were obtained at 0 h, 48 h and 96 h, and the wound width was measured at 48 h. (C) The E. coli-induced protein level of occludin in IPEC-J2 cells after pretreatment with MPX was determined by western blotting. (D) The effect of MPX on the expression of occludin in IPEC-J2 cells was assessed by confocal laser microscopy. (E) MPX increases TEER of differentiated IPEC-J2 cells monolayers subjected to E. coli stimulus. Error bars represent mean ± SEM, n = 3. Statistical significance was defined as follows: #P < 0.05; ##P < 0.01; ###P < 0.001 E. coli vs control; *P < 0.05; **P < 0.01; ***P < 0.001 MPX treatment vs E. coli.

TJs are the structures that connecting adjacent epithelial cells, which have a sealing effect on the intercellular space and prevent toxic and harmful substances in the intestinal cavity from entering the submucosa through the epithelial cell gap. TJs are composed of a variety of TJ proteins, including ZOs, occludin, and claudins (Martens et al., 2020). To evaluate the effects of MPX after *E. coli* infection, the expression of ZO-1, occludin, and claudin-1 was determined by real-time PCR. As shown in Figure 2A, the *E. coli*-induced mRNA expression of ZO-1 and occludin in IPEC-J2 cells was significantly increased after MPX treatment (*p* < 0.05), while the expression of claudin-1 was not significantly altered (*p* > 0.05). Interestingly, in a wound healing assay, the wound width was significantly reduced at 48 h in IPEC-J2 cells treated with MPX (Figure 2B, *p* < 0.01), indicating that MPX is beneficial for healing intestinal epithelial cell damage. Furthermore, western blot and immunofluorescence analyses were used to investigate the effects of MPX on TJ proteins in IPEC-J2 cells after *E. coli* infection. As shown in Figures 2C,D, compared with the control treatment, MPX significantly increased the expression of occludin in IPEC-J2 cells. Moreover, the expression of occludin induced by *E. coli* was significantly increased after treatment with MPX. These results suggested that MPX could significantly increase the TJ protein expression inhibited by *E. coli* in IPEC-J2 cells. TEER is a key parameter of epithelial and used as a measure of cell monolayer integrity. The effect of MPX on *E. coli*-induced intestinal permeability was detected by TEER. As shown in Figure 2E, *E. coli* induced significantly the decrease of TEER continuing to 24 h. In contrast, 10 μg/ml MPX alone increased TEER, indicating that MPX reduced intestinal epithelium permeability. Co-treatment of MPX and *E. coli* demonstrated that MPX repaired *E. coli*-induced intestinal TJ permeability in IPEC-J2 cells. Thus, MPX is able to protect *E. coli*-induced intestinal permeability.

### MPX Protects Mice From Fatal *Escherichia coli* Infection

**Figure 3:**
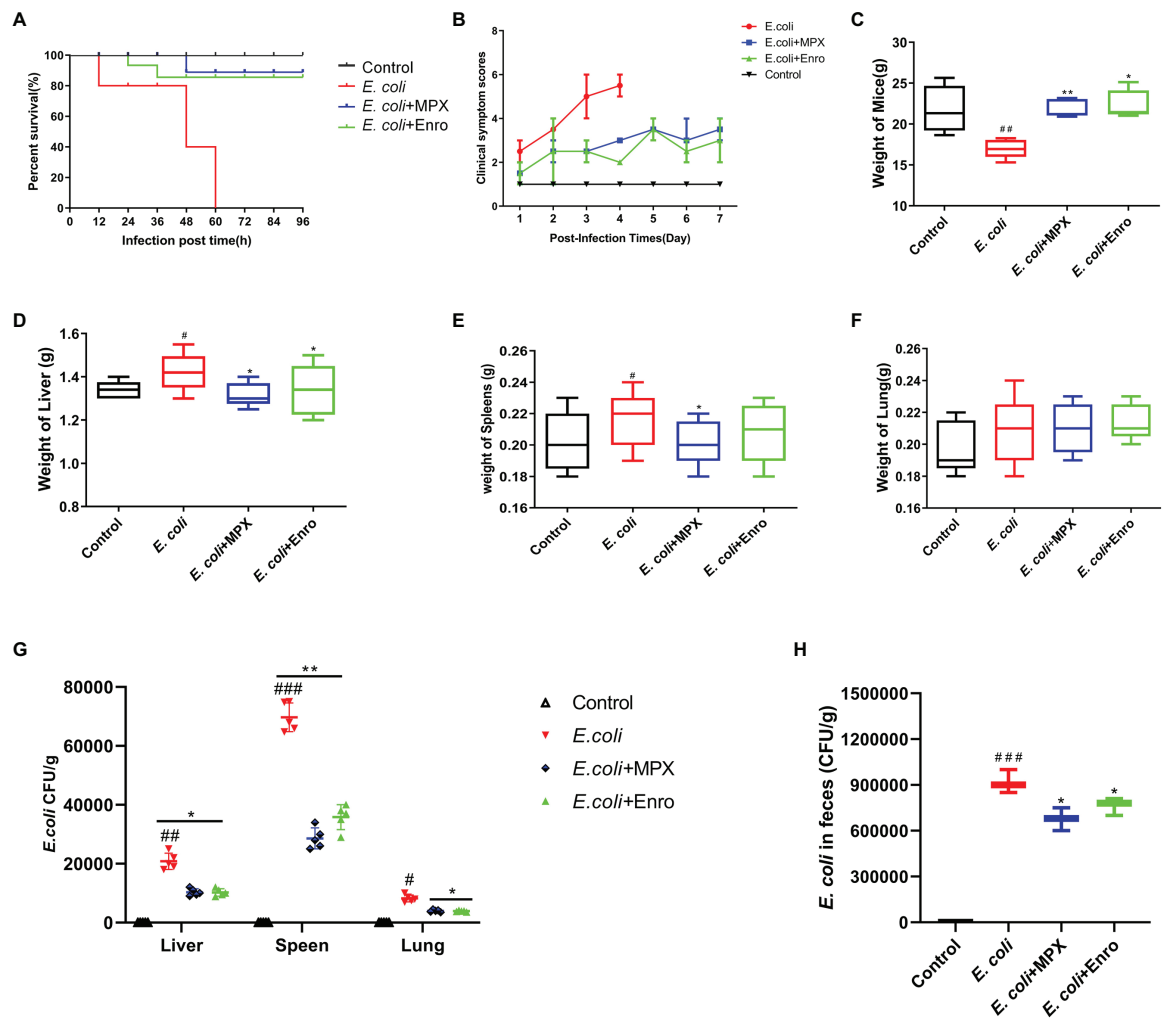
MPX protects mice against infection with a lethal dose of E. coli. (A) The survival rate of mice infected with E. coli after MPX treatment. (B) The clinical symptom score of mice infected with E. coli after MPX treatment. (C) The weight of mice infected with E. coli after MPX treatment. (D-F) The weight of the liver, spleen and lung of mice infected with E. coli after MPX treatment. (G) The number of bacteria in the liver, spleen and lung of mice infected with E. coli after MPX treatment. (H) The number of bacteria in the feces of mice infected with E. coli after MPX treatment. Control group, mice injected with sterile saline; E. coli group, mice injected with E. coli; MPX group, mice treated with intraperitoneal injection MPX and injected with E. coli. Error bars represent mean ± SEM, n = 5. Statistical significance was defined as follows: #P < 0.05; ##P < 0.01; ###P < 0.001 E. coli vs control; *P < 0.05; **P < 0.01; ***P < 0.001 MPX and Enro treatment vs E. coli.

The therapeutic effect of MPX on intestinal inflammation and the intestinal barrier was evaluated in a BALB/c mouse model. The results showed that MPX could protect the mice from a lethal dose of *E. coli*, and the survival rate of the mice was 90%; this effect of MPX was better than that of Enro. However, all the mice were infected with *E. coli* without MPX treatment within 60 h (Figure 3A). The observation of clinical symptoms revealed that *E. coli* infection caused severe diarrhea, lack of energy, loss of appetite, clustering, and messy back hair, while these symptoms were significantly alleviated after MPX treatment; these effects of MPX were superior to those of the same dose of Enro (Figure 3B). The weights of the mice infected with *E. coli* were notably reduced (Figure 3C, *p* < 0.01) but were significantly increased after MPX and Enro treatment (Figure 3C, *p* < 0.01) and were not significantly different from those of control mice (Figure 3C, *p* > 0.05). The weights of the livers and spleens in the *E. coli* infection group were notably high (Figures 3D,E, *p* < 0.05) and were significantly decreased after MPX treatment; after MPX treatment, these weights were not significantly different from those in the control group (Figures 3D,E, *p* > 0.05). The lung weights were not significantly different after *E. coli* infection (Figure 3F, *p* > 0.05). The colonization of *E. coli* in the mouse liver, spleen, lung, and feces was examined by counting on LB agar plates. The number of bacteria colonizing the spleens of mice in the *E. coli* group was greater than those colonizing the liver and lung, and this number was significantly decreased after MPX and Enro treatment (Figure 3G, *p* < 0.05). The number of bacteria colonizing the feces was significantly lower after MPX and Enro treatment than after infection with *E. coli* alone (Figure 3H, *p* > 0.05). These results indicated that MPX exerted good antibacterial effects *in vivo* and protected against lethal infection with *E. coli* in mice.

### MPX Reduces Inflammatory Cytokine Expression and Improves Intestinal Morphology

**Figure 4:**
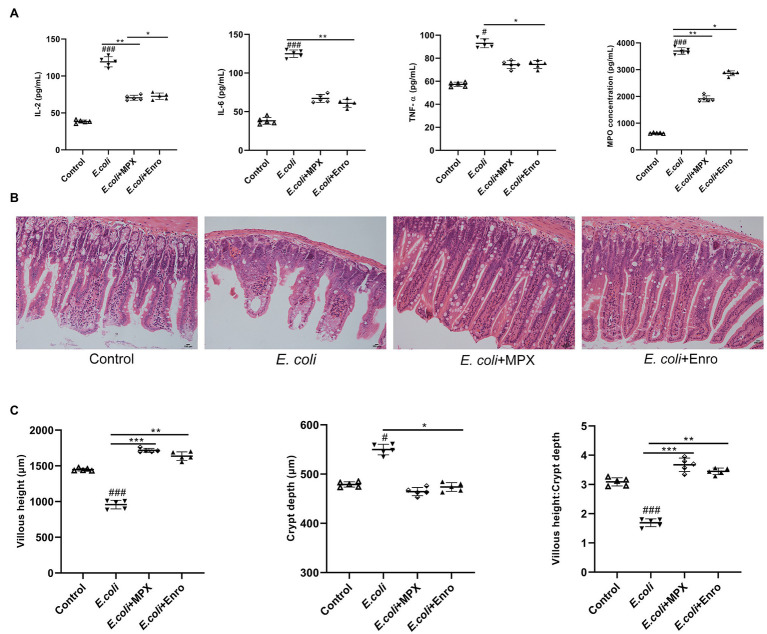
MPX inhibits inflammatory cytokine expression and improves intestinal morphology. (A) The levels of inflammatory cytokines (IL-2, IL-6 and TNF-α) and MPO in mouse serum were detected using ELISA. (B) The jejunum of mice was stained with H&E (bars, 100 μm); images were obtained at 200× magnification. (C) The length of jejunal villi, the depth of crypts, and the ratios of villi length to crypt depth were detected by ipwin32 software. Error bars represent mean ± SEM, n = 5. Statistical significance was defined as follows: #P < 0.05; ##P < 0.01; ###P < 0.001 E. coli vs control; *P < 0.05; **P < 0.01; ***P < 0.001 MPX and Enro treatment vs E. coli.

MPO activity is an index of neutrophil infiltration and inflammation, and MPO can produce specific oxidative species (Malecki et al., 2020). To evaluate the effect of the MPX-mediated anti-inflammatory response after *E. coli* infection, the levels of IL-2, IL-6, TNF-α, and MPO were detected by ELISA. As shown in Figure 4A, the levels of the inflammatory factors IL-2, IL-6, TNF-α, and MPO were significantly increased after *E. coli* infection, while MPX reduced the serum levels of IL-6 (*p* < 0.01), IL-2, TNF-α, and MPO (*p* < 0.05). H&E staining was used to explore the effect of MPX on the intestinal morphology of the jejunum in mice infected with *E. coli*; infection with *E. coli* caused typical intestinal inflammation and barrier damage, shortened villi, reduced mucosal thickness, necrosis, large amounts of inflammatory cell infiltration into the jejunum, and disruption of intestinal villi. MPX treatment increased the villous height and goblet cell counts and decreased the infiltration of leukocytes into the jejunum, and these levels were not significantly different from those in the control group (Figure 4B). Moreover, compared with *E. coli* infection alone, MPX treatment increased the villus length in the mouse jejunum, decreased the crypt depth, and increased the ratio of villus height to crypt depth, and these effects of MPX were better than those of Enro (Figure 4C, *p* < 0.05). These results suggested that MPX effectively reduced the inflammatory factor secretion and improved the intestinal morphology and integrity in mice infected with *E. coli*.

### MPX Improves Intestinal Villi and Microvilli

**Figure 5:**
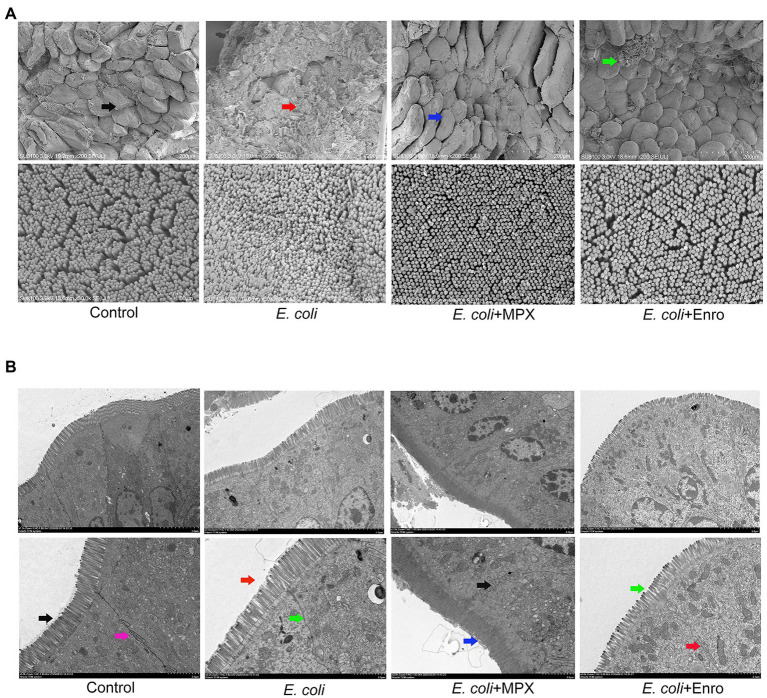
MPX improves the intestinal morphology of the jejunum and the microvilli of intestinal epithelial cells. (A) Morphological changes in the jejunum villi were observed by SEM (upper, 200×; lower, 30,000×). (B) Morphological changes in the microvilli and tight junction proteins in intestinal epithelial cells were observed by TEM (upper, 1500×; lower, 3000×).

Previous H&E staining studies have shown that *E. coli* infection can damage the intestinal morphology of the jejunum in mice. SEM and TEM were used to further evaluate the effects of MPX on the intestinal morphological changes induced by *E. coli*. The SEM results showed that *E. coli* infection severely damaged the morphology and integrity of intestinal villi, while MPX treatment obviously alleviated the injury to the jejunum villi and microvilli, as observed at high (200×) and low (30,000×) magnifications (Figure 5A). We further evaluated the effect of MPX on the microvilli and TJ proteins of intestinal epithelial cells by TEM. *Escherichia coli* infection caused microvilli to fall off, decreased the number of microvilli, and damaged the TJ structure of the intestinal epithelial cells, while MPX treatment increased the number of the microvilli in intestinal epithelial cells; this effect of MPX was better than that of Enro (Figure 5B). These results indicate that MPX can protect against *E. coli*-induced damage to jejunal villi and microvilli in intestinal epithelial cells.

### MPX Suppresses Intestinal Inflammation by Downregulating the Expression of P-P38 and P-P65

**Figure 6:**
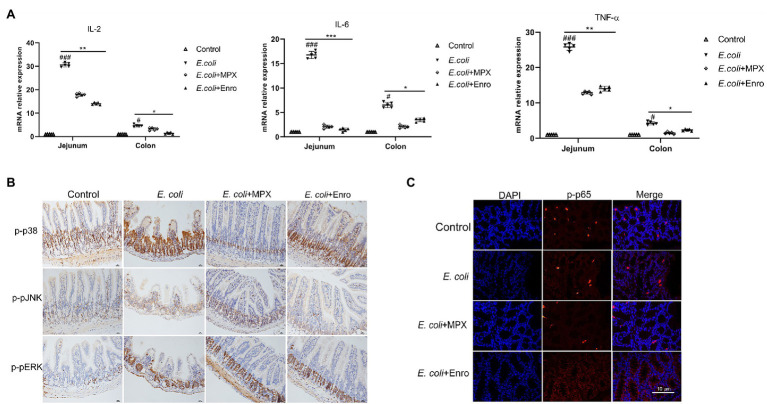
MPX suppresses intestinal inflammation by inhibiting the activation of the MAPK and P65 signaling pathways. (A) The mRNA expression of IL-2, IL-6, and TNF-α in the jejunum and colon after MPX treatment was measured by real-time PCR. (B) The expression of p-p38, p-pJNK and p-pERK in the jejunum after MPX treatment was determined by immunohistochemistry (bars, 100 μm). (C) The effect of MPX on the expression of p-p65 in the colon was assessed by immunofluorescence. Error bars represent mean ± SEM, n = 5. Statistical significance was defined as follows: #P < 0.05; ##P < 0.01; ###P < 0.001 E. coli vs control; *P < 0.05; **P < 0.01; ***P < 0.001 MPX and Enro treatment vs E. coli.

Previous studies showed that MPX treatment could reduce the serum levels of inflammatory factors after *E. coli* infection. To further investigate the anti-inflammatory effect of MPX on the intestine, the mRNA expression of IL-2, IL-6, and TNF-α in the jejunum and colon was detected by real-time PCR. *Escherichia coli* infection significantly increased the expression of the inflammatory factors IL-2, IL-6, and TNF-α in the jejunum and colon (Figure 6A, *p* < 0.01), while MPX and Enro treatment significantly inhibited their mRNA expression in the jejunum (*p* < 0.01) and colon (*p* < 0.05); after MPX and Enro treatment, the levels were not significantly different from those in the control group (*p* > 0.05). Mitogen-activated protein kinase (MAPKs), including JNK, ERK1/2, and p38, are a group of serine/threonine proteins and are involved in the final step of cytoplasmic signal transduction pathways that are activated by multiple extracellular signal pathways. These proteins play a role in the activation of nuclear transcription factor p65, regulating gene expression, and participating in cytokine secretion and apoptosis after activation (Wang et al., 2019b). Immunohistochemistry and immunofluorescence were used to further explore the mechanism by which MPX inhibits the secretion of inflammatory factors. The immunohistochemistry results showed that MPX notably reduced the expression of p-p38 in the crypts of the jejunum, and this effect was superior to those of Enro at the same dose (Figure 6B). However, the expression of p-pJNK and p-pERK in the jejunum was not significantly changed after treatment with MPX and Enro (Figure 6B). These results were consistent with those shown in Figure 1F. In addition, the activation of p65 was analyzed by immunofluorescence, revealing that MPX significantly decreased the phosphorylation of p65 compared with that in the group infected with only *E. coli* (Figure 6C).

### MPX Enhances the Expression of Intestinal Tight Junction Proteins and Mucin

**Figure 7:**
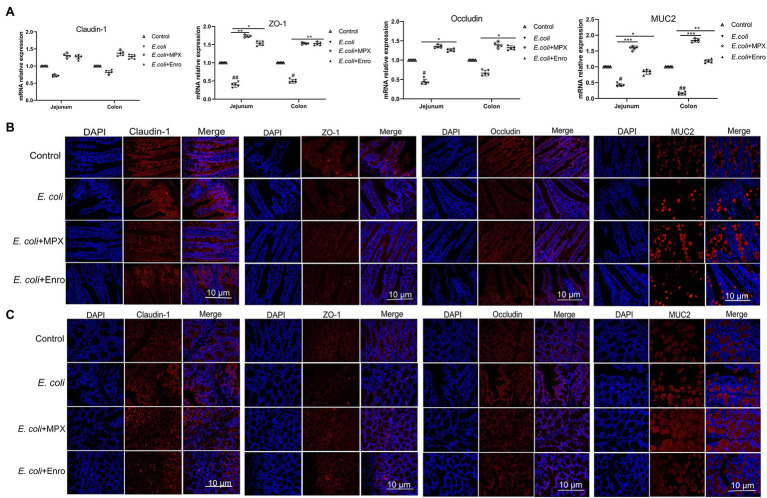
MPX improves the expression of tight junction proteins and mucin in the jejunum and colon. (A) The mRNA expression of claudin-1, ZO-1, occludin and MUC2 in the jejunum and colon. (B) The protein expression of claudin-1, occludin, ZO-1 and MUC2 (red) and DAPI (blue) in the jejunum. (C) The protein expression of claudin-1, occludin, ZO-1 and MUC2 (red) and DAPI (blue). Scale bar = 10 μm.

Mucin is the key matrix-forming component of mucus, which is an innate protective barrier that protects the host from pathogenic attack (Lutz et al., 2020). Analyses of IPEC-J2 cells showed that MPX could improve TJ protein expression after *E. coli* infection. Immunofluorescence and real-time PCR were used to further investigate the effects of MPX on *E. coli*-induced TJ protein and MUC2 expression in the jejunum and colon. *E. coli* infection decreased the expression of ZO-1, occludin, and MUC2, while MPX treatment significantly increased their expression in the jejunum and colon; this effect of MPX was superior to that of Enro (Figure 7A, *p* < 0.05). However, none of the groups showed a significant effect on the expression of the TJ protein claudin-1 (Figure 7A, *p* > 0.05). Immunofluorescence was used to further study the effect of MPX on TJ protein and MUC2 expression after *E. coli* infection. *Escherichia coli* infection reduced the expression of ZO-1, occludin, and MUC2 in the mouse jejunum and colon, while the expression of ZO-1, occludin, and MUC2 was improved after treatment with MPX. The effect of MPX was better than that of Enro, and the expression levels were not significantly different from those in the control group (Figures 7B,C). Collectively, these results indicate that MPX treatment could significantly improve the expression of TJ proteins and mucin in the jejunum and colon.

## Discussion

Antimicrobial peptides are considered to be the best substitutes for antibiotics due to their beneficial antibacterial, anti-inflammatory, and immune regulatory effects and have become a focus of research in recent years (Mahlapuu et al., 2016). MPX belongs to the mastoparan family, contains 14 amino acids, is highly concentrated in wasp venom, and has good antimicrobial activity against bacteria, indicating that it can be used as an antibiotic substitute in the treatment of bacterial infection (Henriksen et al., 2014). In this study, MPX exhibited almost no cytotoxicity in IPEC-J2 cells, significantly reduced the expression of IL-6 and TNF-α induced by *E. coli*, increased the expression of ZO-1 and occludin in intestinal epithelial cells, and promoted the wound healing of intestinal epithelial cells. The therapeutic effect of MPX was evaluated in a murine model, revealing that MPX could protect against lethal infection with *E. coli* in mice and reduce the expression of the inflammatory factors IL-2, IL-6, and TNF-α, thereby alleviating intestinal inflammation. In addition, MPX improved the intestinal morphology and enhanced the intestinal barrier function. This study evaluated the effect of MPX on *E. coli* infection *in vitro* and *in vivo*, laying a foundation for the treatment of intestinal diseases with MPX.

Previous studies have found that the morphological integrity of villi and microvilli, which play key roles in the absorption of intestinal nutrients, is an important indicator of the performance and health of the host (Csernus and Czeglédi, 2020). Daneshmand et al. (2020) investigated the effect of the addition of the antimicrobial peptide cLFchimera (20 mg/kg) to the diet of broiler chickens in the context of necrotic enteritis (NE) challenge and found that cLFchimera ameliorated intestinal lesions and changes to the villus morphology in the jejunum. Liang et al. (2020) found that bovine antimicrobial peptide-13 (APB-13) had good antiviral activity against transmissible gastroenteritis virus (TGEV) and significantly reduced the piglet diarrhea induced by TGEV, thereby improving the intestinal villus morphology. Previous studies demonstrated that the antimicrobial peptide MccJ25 could protect against enterotoxigenic *E. coli* (ETEC) infection and significantly alleviate the destruction of intestinal morphology and changes in villus morphology in mice infected with ETEC (Ding et al., 2020). Wang et al. (2019a) investigated the effect of the antimicrobial peptide JH-3 on the intestinal inflammation induced by *Salmonella* CVCC541 and found that it could effectively alleviate the pathological damage to the duodenum and jejunum, reduce the loss of intestinal villi, and improve the morphology of intestinal villi. In this study, MPX significantly improved the pathological damage to the intestine caused by *E. coli*, reduced the loss of intestinal villi, and maintained the morphology of intestinal villi. The effects of MPX on the intestinal epithelial cell microvilli were further confirmed by TEM, revealing that MPX not only improved the morphology of intestinal villi but also increased the number of microvilli, thereby increasing nutrient absorption in the intestine. These results indicated that MPX could effectively alleviate intestinal damage and maintain villi and microvilli morphology, thereby promoting nutrient absorption in the intestine.

Antimicrobial peptides, as an important part of the natural immune system, possess good anti-inflammatory activity (Wang et al., 2020). Long-term and excessive production of proinflammatory cytokines may lead to intestinal damage and high energy requirements (Lee et al., 2013). Wubulikasimu et al. (2020) found that the antimicrobial peptide AKK8 possessed good antibacterial activity against drug-resistant strains of *Candida albicans*, significantly reducing the levels of IL-6, IL-1β, and TNF-α in the serum of mice infected with *C. albicans* (Wubulikasimu et al., 2020). Ding et al. (2020) evaluated the effect of the antimicrobial peptide microcin J25 on ETEC infections in a murine model and found that microcin J25 decreased the secretion of inflammatory factors by inhibiting the activation of the MAPK and NF-κB signaling pathways, thereby alleviating the intestinal inflammatory response induced by ETEC. Shin et al. (2020) investigated the effect of the antimicrobial peptide Lycotoxin-Pa4a on the lipopolysaccharide (LPS)-induced inflammatory response in RAW264.7 cells and found that it significantly reduced the expression of the inflammatory cytokines IL-1β and TNF-α by inhibiting the activation of the MAPK pathway, thereby inhibiting the LPS-induced inflammatory response in RAW264.7 cells. In this study, MPX significantly reduced the *E. coli*-mediated expression of the inflammatory factors IL-2, IL-6, and TNF-α by inhibiting the activation of p-p38 and p-p65 *in vitro* and *in vivo*, thereby attenuating intestinal inflammation. These results indicated that MPX exerts good anti-inflammatory effects and can be beneficial as an alternative to antibiotics.

The intestinal barrier mainly includes the intestinal epithelial barrier, immune barrier, chemical barrier, and biological barrier. In addition, the intestinal epithelial barrier is the first barrier for preventing bacteria, antigens, and other toxic and harmful substances from entering the submucosa of the intestine and blood (Han et al., 2015). The TJ structure is composed of TJ proteins, such as ZO-1, occludin, and claudins, which are an important part of the intestinal epithelial barrier and play important roles in intestinal epithelial cells (Huang et al., 2020). Lin et al. (2019) investigated the effect of the antimicrobial peptide gloverin A2 (BMGlvA2) on ETEC-induced intestinal barrier disruption in mice and found that it clearly improved the expression of the TJ protein ZO-1 in the intestine after ETEC infection. Zhang et al. (2019) found that the hybrid peptide LL-37-Tα1 (LTA) could increase the LPS-induced expression of ZO-1 and occludin in the jejunum of mice, thereby improving intestinal barrier function. Yu et al. (2020) explored the therapeutic effects of the recombinant antimicrobial peptide microcin J25 on epithelial barrier dysfunction in a murine model and found that it could enhance the expression of TJ proteins, thereby attenuating ETEC-induced intestinal barrier dysfunction. In this study, MPX improved the expression of the TJ proteins ZO-1 and occludin in IPEC-J2 cells and enhanced the expression of ZO-1, occludin, and MUC2 in the mouse jejunum and colon, indicating that it attenuates intestinal barrier dysfunction by improving TJ protein and mucin expression. Surprisingly, MPX was more effective in increasing TJ protein and mucin expression than Enro, which was potentially due to broad antibacterial activity of MPX and antibiotics, which control the balance of microorganisms in the intestine. However, this study of *E. coli* infection by intraperitoneal injection has certain drawbacks and cannot fully represent intestinal infection with *E. coli* strain.

## Conclusion

**Figure 8:**
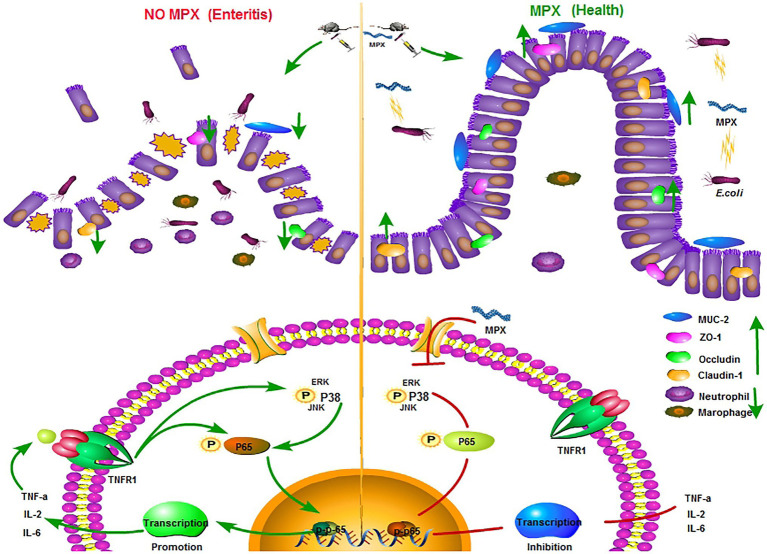
MPX regulates epithelial cells and in vivo signaling pathways.

In summary, as shown in Figure 8, we demonstrated that MPX could reduce *E. coli* growth, attenuate the inflammatory response and intestinal damage, inhibit *E. coli*-induced TLR4 expression, and decrease the IL-2, IL-6, and TNF-α levels by blocking the activation of the p65 and p38 inflammatory pathways *in vitro* and *in vivo*. In addition, MPX improved the intestinal barrier function and increased the expression of the TJ proteins ZO-1, occludin, and mucin. These findings suggest that MPX has excellent antimicrobial and anti-inflammatory activities and is therefore capable of providing protection against pathogen infection, laying the foundation for developing MPX as a substitute for conventionally used antibiotics and drugs. While we herein evaluated the effects of MPX on the intestinal barrier and inflammation in mice, the detailed mechanism of action needs to be further studied.

## Data Availability Statement

The original contributions presented in the study are included in the article/supplementary material, and further inquiries can be directed to the corresponding authors.

## Author Contributions

LW and JH conceived the idea for this study. CZ, XX, SZ, YW, and HZ were involved in the conception and design of the study. XZ performed the experiments and analyzed and interpreted the data. HF, GZ, YB, YX, SC, and JJ were involved in drafting. SL, YW, and XW revised the manuscript. All authors contributed to the article and approved the submitted version.

### Conflict of Interest

The authors declare that the research was conducted in the absence of any commercial or financial relationships that could be construed as a potential conflict of interest.

## Abbreviations

MPX: Antimicrobial peptide MPX
*E. coli*: Enterohemorrhagic *Escherichia coli* O157:H7 ATCC43889
Enro: Enrofloxacin
IL-2: Interleukin-2
IL-6: Interleukin-6
TNF-α: Tumor necrosis factor-α
LDH: Lactate dehydrogenase
MPO: Myeloperoxidase
MUC2: Mucin 2
SEM: Scanning electron microscopy
TEM: Transmission electron microscopy
H&E: Hematoxylin and eosin
